# Barriers to Utilizing Non-replacement Male Calves in the Australian Dairy Industry: A Qualitative Study

**DOI:** 10.3389/fvets.2021.800388

**Published:** 2022-01-27

**Authors:** Veronika Vicic, Anthony J. Saliba, Michael A. Campbell, Jane C. Quinn

**Affiliations:** ^1^School of Agricultural, Environmental and Veterinary Sciences, Charles Sturt University, Wagga Wagga, NSW, Australia; ^2^Graham Center for Agricultural Innovation, Charles Sturt University, Wagga Wagga, NSW, Australia; ^3^School of Psychology, Charles Sturt University, Wagga Wagga, NSW, Australia

**Keywords:** bobby calves, non-replacement male calf, dairy, euthanasia (active voluntary), dairy beef production, beef value chain, producer perceptions

## Abstract

Male non-replacement calves in dairy systems represent an underutilized economic resource for dairy producers worldwide. Despite this, increasing the practice of rearing non-replacement male calves has significant barriers both in on-farm adoption and practice. Poor neonatal rearing practices, higher levels of morbidity and mortality, and disaggregated production pathways with multiple points of handling, have all been described as barriers to adoption of surplus calf production. To identify the critical decision-determining challenges associated with broader adoption of raising non-replacement stock, and to investigate the whole-of-value chain issues faced by dairy producers to rear non-replacement male calves, we undertook a series of semi-structured interviews with Australian dairy producers to interrogate their key challenges. To achieve this, a constructivist grounded theory approach was used to inform the process of analysis of in-depth interviews with Australian dairy producers regarding their current practices and perceptions. Five major themes emerged from these conversations that were key barriers to on-farm non-replacement calf rearing in the producer group participants. These were: impacts of drought on cost and availability of feed for these calves and the whole herd; the management requirements of non-replacement male calves as an additional workload to that of their current operation; their attitudes and current practices to and surrounding euthanasia; perceived ease of supply-chain access for these calves, and their perceptions of the economic value of dairy-beef product as a return on investment. Understanding the barriers to adoption of non-replacement calf rearing, and addressing the value proposition for dairy beef, can assist increased uptake of non-replacement calf rearing. These findings will allow development of strategies to address these barriers, and extension of viable management strategies to increase adoption of profitable business practices surrounding non-replacement male calf production.

## Introduction

There is currently a paucity of knowledge of the practices and management strategies, for animal growth pathways and available markets for non-replacement male calves in the Australian dairy industry. The term “bobby calf” is widely used in Australia as a description of a male calf under six weeks old that is unaccompanied by its dam and the perceived common practice for management of these animals is for them to be slaughtered at < 10 days of age on farm (1). Unlike beef calves that are reared by their dams, in order to maximize milk collection, dairy farmers must artificially rear male calves if they are going to be reared for sale, this representing a major on-farm investment in facilities, feed and time as calves are often housed individually (2) and require relatively high levels of neonatal health management (3–5). As such, and without clear pathways for sale, the economic viability of this practice is often questioned by the industry, making adoption of on-farm rearing a challenge.

There are several production challenges associated with the rearing of non-replacement male calves for beef. These include the need for a protected environment due to their relative immaturity compared to calves weaned under standard conditions who will spend several months with their dams (6). Early-separated dairy calves are more sensitive to climate and other environmental conditions due to their size and age (1) and thus require shelter to maximize their growth and minimize risk of disease. Internationally, the transport of young calves, heat or cold stress, and transit through sales yards have all been shown to cause increased risk of mortality in dairy calves, impacting on producer returns (4, 5, 7). Some specialized producers have established a specific market for rearing non-replacement male dairy calves, but these are not widespread or common. Other perceived deterrents internationally to non-replacement male calf production are perceptions around a lack of obvious saleable markets (8), the perception of the replacement calf as a “low value byproduct” (9) and the limited number of rearing facilities for non-replacement male calves available to take non-replacement male calves for rearing (10). These compounding issues have resulted in the production of non-replacement male calves being identified as a “health and welfare challenge” internationally (8) and a “wicked problem,” namely a problem that is subject to real world constraints and with potentially multiple solutions, for the dairy industry in Australia (11).

Australia is in the minority of developed countries that still perceives the practice of slaughtering non-replacement male dairy calves as more profitable than rearing them for meat production. Despite the perceived practicality, this practice comes with significant welfare implications (9, 12). There are strong indications, both from industry, the public and consumers, that this practice is undesirable with the UK moving to ban this practice by 2023 (13). Globally, consumer opinion is driving practice change (14), with the general perception that calves should be productive as vealers, or slaughtered as mature cattle sold as “dairy beef” (15). Overcoming this issue has been describes as “inherently complex” due to evolving social culture, no ultimate defined solution, stakeholder expectations and producers achieving desired production goals (11). Therefore, understanding producer limitations and adoption of novel practices to manage male calves should present an economically viable option to retain these calves in the beef supply chain.

Although exact numbers of dairy-produced calves born in Australia are not known, recent figures suggest that approximately 400,000 non-replacement calves are processed each year in Australian abattoirs, with this number increasing from 2010 to the present (16). This number of non-replacement calves could represent a valuable proposition to dairy producers if they were to be utilized in an economically viable manner (17). To promote viable production practices for surplus male dairy calves, there is a need to define the barriers that are unique to the Australian dairy industry surrounding the adoption of non-replacement male-calf rearing and the generation of a profitable dairy-beef value chain.

To better understand the perceptions and challenges of Australian dairy producers in relation to adoption of rearing non-replacement calves for beef production we sought to investigate current producer experience of on-farm rearing strategies for non-replacement dairy calves and their associated challenges. Factors of interest included accessibility to markets for non-replacement male calves and dairy producer's perceptions surrounding dairy-beef products in relation to marketability and eating quality. A qualitative methodology with semi-structured interview questions was used to provide impromptu questions to suit the individual producer's responses. This allowed the researchers to capture the range and breadth of producer perceptions to current barriers to adoption of a viable dairy beef supply chain in Australia in the context of their individual enterprises.

## Methods and Materials

Ethical approval for the collection of original data from human participants for the interviews reported in this manuscript was provided by Charles Sturt University Human Research Ethics Committee (Protocol number: H19225). All participants gave informed consent to participate in these interviews. This work was carried out in full compliance with the National Statement on Ethical Conduct in Human Research (2007, updated 2018) and in accordance with the National Health and Medical Research Council Act (1992).

### Methodological Framework

Semi-structured in-depth interviews were conducted with Australian dairy producers. Constructivist Grounded Theory (CGT) informed the research processes and analysis undertaken (18). In this study, the use of CGT as a methodology (19) allowed the researchers to explore, develop knowledge and focus on subjective experiences, perceptions and attitudes of participants concerning current issues associated with non-replacement male calves. The interactions, interpretations and understandings from the research allowed the researcher to deduct or build theory based on previous knowledge (19). This epistemology suggests that the researcher's existing knowledge, perceptions, and formal training in the field of dairy production influenced data collection and the interpretations of participant responses (18). The outcomes in turn reflect the researcher's ability to capture the experiences and opinions of producers through participant interactions. The results of the study are therefore a combination of the contribution of the researcher and the participants; the method is therefore able to capture outcomes that quantitative surveys can sometimes miss.

### Sampling Strategy and Participants

Purposeful sampling was adopted to recruit current Australian dairy owners and/or managers over the age of 18 (20). This strategy identified information-rich participants that shared common attributes to provide in-depth knowledge that later formed central themes aligned with the objectives of the study. Aligned attitudes and opinions expressed in the interviews removed extreme variation among participant responses (20). Participant recruitment was achieved by distributing electronic flyers via dairy consultants, domain experts and dairy discussion groups. Few interviews were opportunistic and participant involvement was sought during a secondary study regarding dairy production. A total of 15 participants were interviewed. All participants owned or co-owned a dairy enterprise and were currently working in the enterprise at the time of the interviews (December 2019 to March 2020).

To address the scope of the research question, a saturation sampling technique was used to determine the number of interviews required to be undertaken (21). A point of saturation is reached in a qualitative study when no new insights or development of novel themes emerge from participant information, and data collection can terminate (22). Saturation was achieved after interviewing 13 participants which provided an information rich dataset enabling the research question to be addressed (20). No new insights were yielded, however a further two interviews were conducted to confirm this assumption.

### Data Collection and Analysis

Fifteen face to face in-depth semi-structured interviews were conducted and audio-recorded by the first author. The interview questions focused on topics that explored past, present and emerging practices associated with rearing non-replacement male calves in dairy systems. A record of practice change (past, present and emerging) over time, allowed the researchers to assess the concurrent attitudes of producers surrounding their responsibility in relation to treatment and welfare of calves. It was of utmost importance to the study to interview owners and/or managers of dairy enterprises as they are the individuals who can implement the greatest changes within each production system. Identification of supply-chains for non-replacement male calves were accounted for through each participant's personal experience regarding the saleability of past male calves and expected future markets. The interview questions were designed to be presented in an open-ended manner. This approach was used to ensure unforeseen comments would be accounted for and subsequent questions could be tailored to each interview allowing overarching themes to remain central to the discussion formulated. Briefly, the interview questions covered the participant's involvement in the dairy industry, the scope of the dairy operation they managed and/or owned, calving management practices with a focus on non-replacement male calves, attitudes and practices toward euthanasia of non-replacement male calves, ideal management strategies of non-replacement male calves and how the strategies could be achieved, and opinions toward dairy beef products. A copy of the interview questions is available from the corresponding author upon request.

The researcher conducted each interview face to face, traveling to the location of each dairy enterprise where the participant resided. Convenience, ensuring participant confidentiality and reducing variation among data collection was a priority of the research team and is why the format of each interview was conducted in this manner. A $20 gift card was offered to each participant as compensation and a token of appreciation for engaging in the study and providing personal insight that contributed to the outcomes of the research.

**Figure 1:**
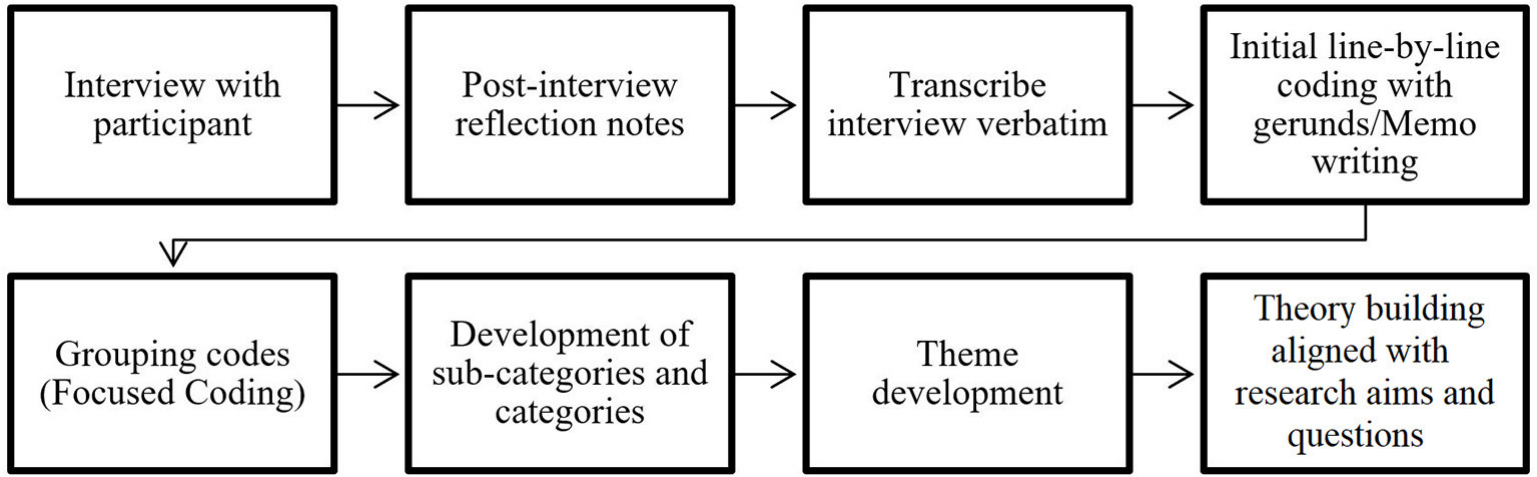
Constructivist grounded theory data collection and analysis process.

Figure 1 depicts the process the researcher undertook to interview and examine the results and develop new findings. Audio recordings from each interview were de-identified and transcribed verbatim by the first author post interview. The author reflected after each interview and adapted and / or added questions accordingly to suit new emerging topics. Sections of audio where the researcher could not comprehend what the interviewee said were noted as inaudible. Each transcript was then proofread. This process lead the researcher to become familiar with the dataset and initiate the process of analysis. The data was hand coded line-by-line with gerunds to allow the researcher to study each fragment of the data and help define meaning, make comparisons and recognize emerging links within the data. Memo writing assisted with the development and reflection of early categories emerging in the data (18, 23). An electronic copy of the coded data was then created on a Microsoft Word document. This process was used to rearrange and segregate codes to establish analytical categorization through focus coding (18). To generate the development of categories and later central themes, a search of supporting evidence through the raw interview data was undertaken to negate or lead to connections between micro and macro levels of the significant themes established (18). The individuality of each participant's experience were linked here to drive novel findings and generated theory aligned to the research aims and questions.

## Results

A total of 15 participants were interviewed. The length of interviews ranged from 15 to 50 min with the average interview length of 22 min. All participants owned or co-owned a dairy enterprise and were currently working in the enterprise at the time of the interviews (December 2019 to March 2020). There were six female participants and nine male participants. All participants were located in south Eastern Australia, ten in the Riverina area of New South Wales and five were located in the Western Districts of Victoria. Herd sizes ranged from 160 to 800 head, with the majority of enterprises having a herd size of 200 to 400 milking cows.

Several major themes emerged: these focused on market sustainability, drought, and resourcing. All participants reported that drought had impacted all facets of their dairy operations, from daily production and management decisions to market access opportunities. All participants also identified they had access to one or more saleable markets for non-replacement male dairy calves in Australia, however, only half of the participants reported supply-chain access that was consistent and economically viable. The remaining participants reported that they frequently had to access opportunistic markets, the majority of which were non-profitable. This cohort of participants did not have a level of confidence in their rearing and sale processes, and commonly resorted to selling calves to the “bobby truck” (a colloquial term for the truck used to transport non-replacement male dairy calves to the saleyards or calf rearers), through the sale yards or social media outlets such as Facebook. Market access was not influenced by location of each dairy enterprise as challenges were seen across both NSW and VIC. All participants reported a range of barriers to rear non-replacement male calves in an economically viable manner including, but not limited to: “drought,” “feed,” “resources,” “space,” “land,” “infrastructure,” “time/labor,” “cost/money,” and “finding a market.” Surprisingly, only two participants, reported the practice of euthanasia of non-replacement male calves on farm, but also stated that if a viable market was available, this route would take priority. For all participants euthanasia was not a preferred practice. These findings accentuate the need for producers to have access to profitable markets to sell non-replacement male calves, and to trust in those systems.

### Primary Themes

Current knowledge, attitudes and practices associated with rearing non-replacement male calves in Australian dairy systems were identified through the subjective experiences of producers. These experiences generated five primary themes in the analysis: (1) impacts of drought, (2) management of non-replacement male calves, (3) euthanasia-related attitudes and practices, (4) supply-chain access, and (5) value of dairy-beef products.

### Impacts of Drought

Statements regarding the impacts of drought on dairy production was prevalent throughout the interview process and as such, became a central topic of conversation. Nine participants reported drought conditions impacted production practices and management choices, which in turn affected all facets of their operation.

Poor seasonal conditions contributed to an increase in the time and labor allocated to monitoring animals, feed allocation, water management as well as presenting reduced market opportunities for all livestock. The additional operational costs required as a consequence of ongoing drought formed a large portion of the participants reflections.

*Producer A: “We didn't calve many this year…consequence of being in 2 years of drought and were down to next to no water allocation…so the last 12 months the numbers have been dwindling down…”*

In contrast, major drought impacts did not affect six participants that had access to bore water, irrigated pasture and feed stockpiles. This cohort of participants were not as conscious of seasonal drought as these provisions were recognized to be alleviated.

*Producer B: “…we store up as much fodder as we can we usually carry about 2 years worth of hay…we still have got enough feed in storage to carry us through but not pushing production…”*

*Producers C: “…the droughts take a huge toll…we are a little bit protected here because we grow all our own feed…but still…usually we have surplus grain…so usually we have that income as well…”*

As such, location-specific impacts of drought were not observed. Within the same geographical region, participants who were better prepared for drought conditions expressed concern for other dairy producers who were not in such a fortunate position. These participants agreed that enduring previous droughts had forced innovation in their operations and promoted increased efficiencies within their production system to protect them against future economic risk associated with drought. Those participants that were more adaptable in their operating practices were at lower risk of encountering economic loss in the face of challenging climate conditions. The degree of focus on innovation differed, with some participants indicating a culture of innovation and others relying on traditional knowledge.

*Producer A: “…trying to be as flexible in your management approach on the farm as you can be, and sustain it…it's not just a matter of this is the way I do it, oh hang on there's a drought, or I've got no water, ring up and buy in a stack of hay and grain…you know question everything you do… well is there a better way…even me sons coming up with new ideas and changing things around…”*

Nine participants reported reduced profit margins and that these reductions were directly correlated with ongoing drought conditions. Factors predicating this economic cost included deteriorating land conditions leading to increased reliance on supplementary feeding and increased labor costs. Poorer quality feed resources resulted in decreased animal body condition scores that resulted in higher rates of morbidity and mortality on farm, reduced sale prices and decreased supply chain access for all on-farm animals, including non-replacement male calves. Notably, the difficulty in selling non-replacement calves and reduction in price at point of sale forced two participants to revert to euthanasia practices where they had been previously able to avoid doing so.

*Producer D: “We never used to [euthanise] but we have had to because of the cost and then the sale yards and sometimes getting five dollars is not worth all that time and milk…”*

### Management of Non-replacement Male Calves

All participants reported that retained heifer calves received vaccinations and any other required veterinary prophylactic treatments as per standard production practices. Retained heifer calves received a consistent feed allocation that was inclusive of milk, *ad libitum* hay and concentrate in the form of grain or pellets. Shelter was also provided to reduce environmental stressors during the critical period of early weaning and growth. The majority of producers interviewed reported that retained heifer calves took priority over non-replacement male calves. Specifically, six producers noted that vaccination and treatment protocols, feed quality and shelter provided to heifer calves was not replicated for non-replacement male calves.

*Producer E: “…if things get a bit tight…you know your guys [non-replacement male calves] are going to have to do it a bit harder than the heifers. Heifers will get first choice on where they go and they'll get grain and better hay…if I have to feed the steers grain, the hay it won't be as good…”*

Four producers did identify that they felt it was their responsibility to treat all calves in a similar manner as a part of their “social license to operate” and to ensure that all animals were cared for in a “reasonable way.” One producer had formal training as a veterinarian and justified this opinion with the explanation that the value of treating male calves maintained generally high health standards across the herd and increased treatment success rate among all calves, inclusive of the heifer calves that were a “long-term investment.”

*Producer C: “…from an experience point of view I have treated a lot of bull calves…it makes me much better at treating the heifers in the same position so I justified that way and I am much better looking after the heifers which you know are more valuable…I just don't like [not treating non-replacement male calves], it's still a life and it's got some value in it…I know that some farmers wouldn't be able to justify the cost of drugs and the extra time but that's my policy here…”*

However, one participant suggested that veterinary treatment of male calves reduced their ability to pass these animals on to saleyards due to the need to comply with industry withholding periods as a justification for their different management strategies between male and female calves.

*Producer D: “…we try and sell them straight away, like a week-old so we can't [send them to a sale yard] if they are treated and at the moment there is…no return for us to chase that market so we don't bother.”*

Producers that did not manage heifer and non-replacement male calves in a similar manner identified “time,” “labor” and “costs” as the major barriers to this differential treatment. They reported that a lot of time is spent “off the books” to facilitate rearing male calves, implying a negative cost benefit to their operation. *One* participant offset the extra labor requirements through the use of a robotic feeding system. Some participants reported inefficient or inadequate physical infrastructure, and/or space restrictions as limiting their ability to rear male calves. In some cases, this was a direct effect of expansion and growth within the milking herd leading to less space to house and rear male calves. Inadequate housing due to space constraints and herd growth often resulted in male calves being housed in exposed pens and/or paddocks, resulting in increased mortality and morbidity rates.

*Producer F: “…deciding to keep the bull calves was a big change because we probably…at the time we had enough room, the calf numbers were a lot lower because our cow numbers were at that low point…but now the numbers are getting big, the calving groups are getting big…that's why they're [non-replacement male calves] in those makeshift kind of pens. …we're currently working on building a calf shed because I think our main problem at the moment is [the non-replacement male] calves exposure to the weather…I think that's when the calves are most susceptible to getting sick. They're freezing cold during the night and then really hot through the day, or the rainy weather…”*

The opposite was true of these producers with available grazing land that appeared to facilitate the rearing of male calves to steers with fewer on-farm limitations.

*Producer B: “It works in quite well because we got a fair bit of hill country, about 900 acres of that. So once we get them off the bucket that's the most labor-intensive part of it…then you only drench them and vaccinate them once and you put them out the back and forget about them so labor-intensive is not there.”*

### Euthanasia Attitudes and Practices

Participant opinions surrounding euthanasia varied, however, all participants agreed that euthanasia of non-replacement male calves should only occur as a last resort. This response included two participants who reported currently practicing euthanasia of non-replacement male calves. Twelve participants reported that they would never euthanise non-replacement male calves, even if it was non-profitable to rear them. These participants indicated strong feelings that the dairy industry must acknowledge non-replacement male calves as a part of every dairy production system and manage the production of male calves responsibly.

*Producer G: “…part of our social license…is to make sure that [we are] caring for all our animals in a reasonable way.”*

*Producer H: “…I see it's more unethical to be slaughtering calves like some people are doing on-farm than selling them to the abattoirs and using that product…whether you look at it from a commercial environment or social [point of view]…why are you killing something…that is perfectly healthy and perfectly edible…it would probably be more economically sensible for us to shoot that calf in the paddock and be done with it…it is economically costing us money sending it to an abattoir but I think that's better use of the resource…”*

These participants expressed several terms with negative connotations when describing their feelings toward male calf euthanasia, such as “avoid,” “frustrated,” “hate,” “not supportive,” “refuse,” “unethical” and “unpleasant.” One participant reported previously practicing euthanasia and expressed high levels of personal frustration toward having to euthanize a healthy calf. Adverse effects on the producer's mental health and well-being were also reported in line with this practice. Specifically, one participant did not allow other farm employees to euthanize calves for this reason. They did, however, identify that formal training in euthanasia reduced the toll on their mental well-being as it provided confidence the practice was conducted in the most humane way.

*Producer A: “It's very frustrating to [euthanise] because you've got a perfectly healthy calf, good fit decent size calf and he is good for nothing, and you go I'm shooting a damn good calf, why? … no one likes putting down good healthy calves so it wouldn't be good for staff's mental health either… before we were trained and got the captive bolt, I would leave stock for my dad to put down…I wasn't confident…when we did the captive bolt training and you're actually equipped to know exactly what you're doing, not just what you were taught by your dad or whatever…when you actually understand the science…it helps…”*

Two participants, one who did euthanize and one who did not, reported that if it was not economical to rear male calves they considered it to be “acceptable” to euthanise non-replacement male calves. It was suggested that, in this instance, the milking herd should take priority for resource allocation. The participant who did practice euthanasia believed more negative impacts and stress was placed on non-replacement male calves sold through sale yards or not reared with optimal management strategies than the practice of humane euthanasia.

*Producer D: “…well at the end of the day it is going to get euthanised isn't it…so I think rather than it getting sick…or going through the stress of the sale yard, I think is the most humane [to euthanise] …we've just got to be realistic about it, operating within our means…”*

*Producer B: “…I don't have an issue with it, we had to do it years ago…if there's not a market there, what you do with them…As long as it's done humanely it's not a problem, it's just something's gotta be done - it's a fact of life…”*

### “Dairy-Beef” Supply-Chain Access

Every participant reported the desire to access better markets for non-replacement male calves and to improve their production management practices but they also identified many constraints. These constraints presented an economic barrier to dairy producers and reduced their ability to make consciousness-based decisions while maintaining a profitable enterprise. This finding was in contrast to their treatment of replacement heifer (female) calves where their practices were highly consistent.

**Figure 2:**
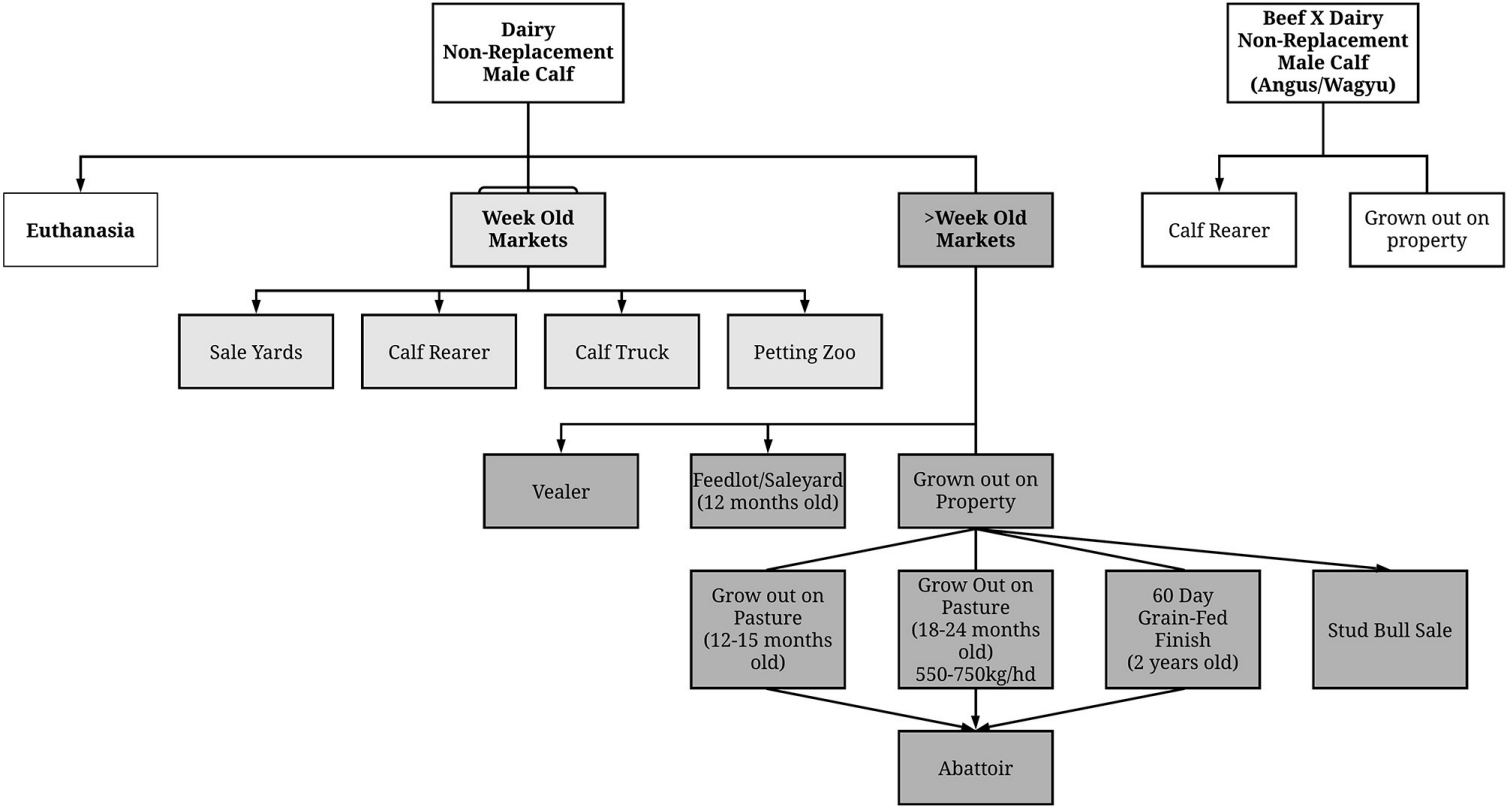
Supply chain access for non-replacement male calves. Arrow (→) in diagram denotes end point and/or point of sale from the property of origin.

A visual representation of the co-relationships between the interview information surrounding producer market access/supply chains for non-replacement male calves was compiled from their statements and is shown in Figure 2. Both dairy breeds, and beef x dairy first cross offspring were considered separately. Dairy breed producers identified three main production pathways for their non-replacement male calves, (1) calves that were sold to market at 7 days of age; (2) calves that were sold to markets at > 7 days of age; and (3) those that were euthanized. Those calves that were retained on farm longer than 7 days showed a greater number of finishing pathways than those sold very young. Beef first cross offspring only identified two pathways to sale: the calf rearer or retained on the home property.

The supply chains identified, and their relative profitability, varied between producers. Some producers reported inconsistent use of target supply chains (sale yards, vealer, steer market) for the sale of non-replacement male calves. This cohort of producers were holding onto their non-replacement male calves until market conditions improved (typically, prices to increase post drought) before committing to sale. These producers were therefore at higher risk of drought associated economic loss by this practice.

*Producer A: “No specific market in mind at this point…at this stage if we break even that'll be about it, I don't expect to make any money on them at the moment, but going forward, better seasons, yes we hope to make a few bucks on them.”*

In contrast, producers who had well-established sale options in place for non-replacement male calves, including pre-contracted sales to calf rearers and/or returning customers, reported successful sales and market access. They also appeared to have a positive outlook on the utility and viability of non-replacement calf production and were more amenable to seeking alternative production solutions.

### Breed Characteristics and Value of a “Dairy Beef” Product

Of the 15 producers interviewed, the majority (9/15) were not using other non-dairy breed genetics to increase likelihood of carcass yield in their male non-replacement offspring. For these producers, their focus was on dairy production and their sires were of primary dairy genetics: these included mixed crosses (Holstein x Jersey; Friesian x Jersey; Friesian x Ayrshire) with Brown Swiss, Montbeliarde and Swedish Red cattle all reported, or pure breed genetics (Friesian, Jersey or Holstein). The remaining six producers reported using beef breed genetics to improve carcass yield with Angus (4) being the predominant breed of choice, the others reporting use of Wagyu and BeefX bulls.

Participant views toward dairy-beef products only emerged as a point of interest during the fifth interview and therefore not all participants expressed an opinion on this topic. Within this sub-cohort of participants (*n* = 11), opinions varied regarding the value of a dairy-beef product. Although the dairy producers were not market experts, they also indicated their perceptions of consumer behavior toward dairy-beef. A latent theme suggested most participants did not believe that dairy-beef could target a premium market. The perceptions of this cohort of participants can be separated into two clear categories: those who thought dairy-beef could not be considered a premium product, and those who considered that there was a premium market for dairy-beef.

*Producer G: “I don't know whether you would…advertise something as “dairy-beef” …you would want to do a lot of research on the name “dairy-beef” to see what connotations come up with it…you could be creating a can of worms for the people that might be euthanising…”*

Three participants believed that beef from dairy animals had similar or inferior meat and eating quality to other beef breeds. One participant conveyed doubt in the supply chain for dairy-produced beef carcasses explaining they currently experienced a price discount at slaughter for their carcasses, an industry implication that this represents a carcass of lesser eating quality. Interestingly, this participant also had a perception that dairy beef carcass and eating quality outcomes were equal to those of conventional beef animals despite the dairy carcass receiving a discount. Another participant agreed that dairy bred animals receive discount at slaughter but suggested that this was due to inferior pre-slaughter nutrition and that these animals would require grain-finishing to perform well.

*Producer I: “…I finished some Friesian steers…on grain for the last 60 days so I got a pretty good price for them…they yielded [well]… but generally in Western Victoria you cop an absolute flogging for Friesians…in terms of you send them to the markets [and] you just get a massive discount even to send to slaughter. Unless you can really finish them off with grain you will get a massive discount, they will pay the lowest end that you can get…”*

Two participants explained their aim was to operate in a similar manner to match the standard they expect when purchasing food for themselves, indicating a high level of food consciousness. These participants were actively restructuring their businesses to suit markets in alignment with their own food preferences. The same producers said they would pay a premium for high-quality food items, including a dairy-beef product. However, they also suggested consumers generally do not think about the treatment of animals when purchasing food items and suggested there was a need to convey a positive animal welfare message for non-replacement male calves as a value chain opportunity. They identified that communication to the public around a dairy-beef product should include the dedication and good stewardship of dairy producers toward non-replacement male calves and their desire to rear them in the best possible manner. Drawing consumer awareness not only to product quality, but also to animal welfare and production, would be essential in creating a viable broad-spectrum dairy-beef brand and market.

*Producer J: “I think you would sell the whole story so really it's going to be about…being clean and green, free range and all that sort of stuff…I'm the one who picks up the bulls…out of the paddock every day and I tell the cows I'm looking after them, they're in my care I have to look after them the best that I can…so I think you have to convey that, it's like a stewardship thing…”*

## Discussion

This qualitative study explored the subjective experiences and attitudes described by dairy producers regarding the production challenges associated with the practice of rearing and growing out non-replacement male calves to sizable beef animals. Although the cohort was relatively small, the methodological approach ensured that identification of alternative views was unlikely due to saturation of responses. The authenticity of in-person interviews to establish the context in which the enterprises were operating was an approach that is more difficult to achieve by phone or internet-based surveys. Five themes characterized each participant's production/management decisions surrounding non-replacement calves: impacts of drought, management of non-replacement male, supply-chain access, euthanasia-related attitudes and practices and value of dairy-beef products.

Within these themes poor profit margins influenced many management choices made by dairy producers; this finding was similar to those observed in other studies internationally where producer concerns for a dairy beef supply chain lay within poor supply chain integration and lack of viable profit margins (8, 24). Dairy beef, through slaughter of cull cows as well as non-replacement calf production accounts for a small, but financially meaningful percentage of a dairy producers income, estimated in the U.S. to be between 5 and 15% of gross income, yet the product from these animals is often not identifiable to the consumer, and attracts downgrading for the producer (25, 26). In Europe, dairy beef production represents approximately one third of all beef produced in this region (27) and is widely accepted by European consumers. Current estimates indicated that approximately 2.8 million head of dairy cattle are processed annually in Australia, representing an important component of the beef and veal industry (28).

In this study, adoption of production of male non-replacement male calves as a viable product for their system were limited by labor, infrastructure, and other resources, resources that were more limited by drought seasons. One potential strategy to support development of an integrated supply chain for dairy beef has been reported by Irish producers when considering resourcing requirements, where grants from government bodies have been used to provide necessary additional infrastructure. This model could aid in preventing euthanasia of non-replacement male calves help to deliver the desired outcomes of the Australian Dairy Sustainability Framework toward reduction of euthanasia of non-replacement calves (24, 29).

Participants in our study reported monetary loss for the sale of non-replacement male calves was generally overcome by arrangement of pre-contracted sales to calf-rearers or other saleable markets (Figure 2). This assisted in giving these producers an economically viable route to market for these animals as oppose to participants who were waiting to see if market conditions improved to increased calf sale profit margins. Irish producers had concerns regarding price volatility and market uncertainty surrounding non-replacement male calves (24), so this represents a common challenge. By utilizing pre-contracted sales for non-replacement male calves, our study shows that this can support a pricing model that guarantees profitability and therefore mitigate risk for the dairy producer. This was also a favored option by Irish producers who preferred to send calves to a rearing facility with pre-contracted prices or retain ownership of calves of those calves in the rearing facility with a pre-contracted price negotiated prior to slaughter (24). This model could allow dairy producers at a national and international level to accommodate the extra labor requirements, facility usage, and grazing land capacity to support rearing of non-replacement animals, allowing more investment into the primary focus of their dairy production whilst supporting an integrated dairy beef supply chain.

Participants in our study reported that they would improve the conditions for rearing of non-replacement male calves if finance was not a potential barrier. This was similarly reported in a cohort of Irish producers where changes or improvement on farm to support a successful dairy beef integration system was the preferred option if finance was not a limiting factor (24). If grants and a more secure pricing structure was available for the sale of non-replacement male calves it could assist producers to invest in improved calf-rearing infrastructure and encourage them to put higher inputs into calf feeding protocols similar to those conditions described by participants for retained heifer calves. Improved feed quality and access to supported housing would assist male calves reaching target weights early and support them in their critical growth periods, improving calf mortality and morbidity rates (1). The interviews undertaken in this study also coincided with a period of sustained drought in south-eastern Australia. Drought was found to exacerbate the financial and practical requirements of non-replacement male calf production due to the cost of fodder, but participants also acknowledged that in a non-drought affected seasons it is currently difficult to make financially viable decisions due to a lack of obvious or easily accessible supply chain options.

Our findings showed that there was not one consistent supply chain preferred by producers interviewed in this study (Figure 2) even within the same geographical region. This suggests that development of a single dairy beef supply chain will be hard to achieve. Previous studies have suggested that multiple production models may be required to give sufficient options to dairy producers to invest in maintaining non-replacement calves for dairy-beef production (24). This may reflect that producers do not know how an integrated model will operate, or that sufficient options are not yet available. This represents a challenge to industry to determine the best dairy beef value chain model for industry adoption through targeted funding.

In our study, participants reported they did not think dairy beef should be marketed as a premium beef product due to “inferior” meat quality traits compared to other traditional beef breeds. This misconception or belief has also been reported in other studies (24, 30). Despite this perception amongst dairy producers, there is a growing body of work that suggests there is no difference between dairy and traditional British beef breeds in growth potential, lean meat yield, yield of prime cuts, and the quality of meat produced when grazed under similar conditions and slaughtered at the same chronological age or the same level of maturity (30). The reasons for this misperception of dairy beef as an inferior product needs to be further investigated. One possible reason could be due to the reported reduction in of quality feed inputs into early dairy-beef production systems from producers in our study, with the perception that this in turn will result in a poorer quality meat product. In contracts to a report by Maher et al. (24), who implied a lack of husbandry skills related to raising non-replacement calves, but rather a choice to treat male calves differently due to preconceived financial disadvantages of retaining these calves in the herd. Equally, perception of price discounts relating to a reduction in eating quality may also be feeding forward into these perceptions. Respondents in Maher et al. (24) also thought they were not receiving a fair price for the amount of labor and time needed for rearing non-replacement calves with this also limiting their desire to continue in production.

Six of the fifteen producers interviewed reported that they were using joining their dairy heifers with traditional beef sires to produce a better performing male non-replacement calf. The most hybrid common crosses were with Angus bulls. These cross-bred calves were seen to be more valuable in the marketplace and received better pricing at slaughter, either from farm or through calf rearers. In a study of Irish producers, Maher (24) reported that Angus, Limousin and Hereford were the sires that Irish dairy producers would consider using to generate dairy beef animals (24, 31). There is evidence to support the perception of Angus as a strong contender for best hybrid carcass production, as studies in New Zealand (31), the United States (32) and Australia (33) have reported that performance, carcass quality and eating quality of crossbred dairy calves sired by Angus bulls was improved compared to dairy sired animals.

Although generally offering reduced yield compared to other dairy breeds (34), Jersey beef has been identified as a particular niche product (32), showing quality traits related to marbling and palatability (35, 36). Interestingly, not many producers in this study utilized Jersey as a breed (only four of fifteen producers interviewed). In contrast to the evidence that the Jersey breed can produce a quality beef carcass (35), our cohort indicated that these male calves were extremely hard to sell, were of least value, and were therefore the commonly euthanized, similar to that reported by Irish producers (24). This suggests that greater education is required on the value proposition of different dairy beef crosses to ensure that the breeding market is informed of the evidence on the relationship between dairy beef genetic composition and carcass quality outcomes. This might improve uptake of more niche diary breeds as a viable genetic cross for high quality dairy beef production if a premium product market could be established.

One of the products of the interviews was that euthanasia of calves was a valid exploratory topic due to the variable access to market options for non-replacement male calves in Australia. Producer statements also identified a key novel finding regarding producer well-being related to experience of non-replacement calf euthanasia in the dairy industry. Euthanasia was recognized as a traumatic experience by some producers and for this reason they were not prepared to delegate this task to other employees. One previous study has identified the practice of euthanasia generating emotional strain in dairy producers (37) and chronic stress associated with euthanasia of animals in other animal professions such as the veterinary industry, has been shown to be related to increased rates of burn out (38). These finding suggests that the human impact of euthanasia of non-replacement dairy calves should also be considered as a key imperative for the generation of viable production pathways for these animals. Dairy producer well-being in relation to production practices is an area that should be further examined in future studies.

## Conclusion

The authors believe this is the first report to examine Australian dairy farmers opinions and attitudes to production and management of non-replacement dairy calves and the dairy beef supply chain. The interviews conducted in this study showed that producers considered there are current challenges to rear non-replacement male calves and that there is a knowledge gap related to optimal practices needed to produce a beef carcass able to meet grid specifications for best return on investment. Finally, the personal impact of performing euthanasia was reported by producers, and was highlighted as a last resort, where other avenues for value-chain integration for non-replacement calves had failed. Clearly, producers are looking for options to maintain these animals as a viable income stream, and more options need to be available either on farm or through other production systems.

In response to these findings, the authors suggest that pricing structures and market stability are segments of the supply chain that could be improved to generate a viable dairy beef supply chain and create future market options for non-replacement calves and their retention in the system. This study will inform future quantitative research to expand on key areas including supply chain markets for non-replacement male calf in Australia and globally. There is also a need to further explore producer well-being related to euthanasia and management of non-replacement male calves in the Australian setting.

## Data Availability Statement

The raw data supporting the conclusions of this article will be made available by the authors on reasonable request.

## Ethics Statement

This study was reviewed and approved by Charles Sturt University Human Research Ethics Committee (Protocol H19225). The participants provided their written informed consent to participate in this study.

## Author Contributions

VV, AS, MC, and JQ conceived and designed the study. VV collected, compiled, analyzed the data, and wrote the first draft of the manuscript. All authors contributed to review of the manuscript and approve the submitted version.

## Funding

VV was supported an Australian Research Training Program Scholarship from Charles Sturt University and a scholarship from the Graham Centre for Agricultural Innovation. MC and JQ are supported by funding from Meat and Livestock Australia.

## Conflict of Interest

The authors declare that the research was conducted in the absence of any commercial or financial relationships that could be construed as a potential conflict of interest.

## Publisher's Note

All claims expressed in this article are solely those of the authors and do not necessarily represent those of their affiliated organizations, or those of the publisher, the editors and the reviewers. Any product that may be evaluated in this article, or claim that may be made by its manufacturer, is not guaranteed or endorsed by the publisher.
